# *Pseudomonas fluorescens* and *Trichoderma asperellum* Enhance Expression of Gα Subunits of the Pea Heterotrimeric G-protein during *Erysiphe pisi* Infection

**DOI:** 10.3389/fpls.2015.01206

**Published:** 2016-01-07

**Authors:** Jai S. Patel, Birinchi K. Sarma, Harikesh B. Singh, Ram S. Upadhyay, Ravindra N. Kharwar, Mushtaq Ahmed

**Affiliations:** ^1^Department of Botany, Banaras Hindu UniversityVaranasi, India; ^2^Department of Mycology and Plant Pathology, Institute of Agricultural Sciences, Banaras Hindu UniversityVaranasi, India

**Keywords:** *Pisum sativum*, *Erysiphe pisi*, G-protein, hydrogen peroxide, stomata, jasmonic acid

## Abstract

We investigated the transcript accumulation patterns of all three subunits of heterotrimeric G-proteins (Gα1 and 2, Gβ, and Gγ) in pea under stimulation of two soil-inhabiting rhizosphere microbes *Pseudomonas fluorescens* OKC and *Trichoderma asperellum* T42. The microbes were either applied individually or co-inoculated and the transcript accumulation patterns were also investigated after challenging the same plants with a fungal biotrophic pathogen *Erysiphe pisi*. We observed that mostly the transcripts of Gα 1 and 2 subunits were accumulated when the plants were treated with the microbes (OKC and T42) either individually or co-inoculated. However, transcript accumulations of Gα subunits were highest in the T42 treatment particularly under the challenge of the biotroph. Transcript accumulations of the other two subunits Gβ and Gγ were either basal or even lower than the basal level. There was an indication for involvement of JA-mediated pathway in the same situations as activation of *LOX1* and *COI1* were relatively enhanced in the microbe co-inoculated treatments. Non-increment of SA content as well as transcripts of SA-dependent *PR1* suggested non-activation of the SA-mediated signal transduction in the interaction of pea with *E. pisi* under the stimuli of OKC and T42. Gα1 and 2 transcript accumulations were further correlated with peroxidases activities, H_2_O_2_ generation and accumulation in ABA in pea leaves under OKC and T42 stimulations and all these activities were positively correlated with stomata closure at early stage of the biotroph challenge. The microbe-induced physiological responses in pea leaves finally led to reduced *E. pisi* development particularly in OKC and T42 co-inoculated plants. We conclude that OKC and T42 pretreatment stimulate transcript accumulations of the Gα1 and Gα2 subunits of the heterotrimeric G protein, peroxidases activities and phenol accumulation in pea during infection by *E. pisi.* The signal transduction was possibly mediated through JA in pea under the stimulus of the microbes and the cumulative effect of the co-inoculated microbes had a suppressive effect on *E. pisi* conidial development on pea leaves.

## Introduction

Understanding the plant immune system is a challenge to the plant biologists. However, a significant understanding had been developed with time in relation to plant’s responses toward various pathogen challenges. Plants respond to biotroph, necrotroph, oomycete, and bacteria differently (Trusov et al., 2010). Involvement of the heterotrimeric G-proteins in plant defense system was first described by Legendre et al. (1992). Unlike in animals, the transmembrane G-protein coupled receptors (GPCRs) are not commonly found in plants and therefore the G-protein subunits (α, β, and γ) are believed to be autoregulated in plants (Urano et al., 2012). However, in a recent report Bommert et al. (2013) demonstrated existence of GPCR like transmembrane receptors in maize that have the ability to activate the Gα subunit for regulation of shoot meristem development. Some other studies in plants have demonstrated direct roles for G-proteins in plant defense against a variety of pathogens (Suharsono et al., 2002; Llorente et al., 2005; Trusov et al., 2006, 2010). But the signaling role and host-pathogen interaction may be specific to each host-pathogen pair and therefore needs thorough investigation to develop a comprehensive understanding on the interaction taking place.

Very few genes were reported which encodes G-protein and involved in different biological processes of plant cell (Jones and Assmann, 2004). While model plant *Arabidopsis* has only one gene for Gα subunit, one for Gβ subunit, and three for Gγ-subunits (Thung et al., 2012), pea has two Gα subunits (Marsh and Kaufman, 1999), rice has two Gγ subunits (Yadav et al., 2012) and *Nicotiana benthamiana* has two Gβ-subunit genes (Zhang et al., 2012). Different subunits of G-protein were involved in disease resistance against a variety of pathogens in different plants. The Gβ was involved in defense against necrotrophic fungi such as *Botrytis cinerea*, *Alternaria brassisicola* and *Plectosphaerella cucumerina*, vascular pathogen *Fusarium oxysporum* in *Arabidopsis*, the Gα-subunit was involved in defense against the hemibiotrophic rice blast pathogen *Magnaporthe grisea* in rice whereas no evidence was found regarding involvement of G-proteins against the oomycete *Peronospora parasitica* and bacterium *Pseudomonas syringae* in *Arabidopsis* (Suharsono et al., 2002; Llorente et al., 2005; Trusov et al., 2006, 2010). The interrelationship between G-proteins and *Arabidopsis* defense responses nevertheless been clearly concluded by Gβγ dimer studies (Urano et al., 2013). The results broadly conclude that the Gα subunit is a negative regulator of defense response against the necrotrophic pathogens while Gβ and Gγ are positive regulators. Demonstrable evidence showed that G-protein mediated defense response include production of reactive oxygen species (ROS) including H_2_O_2_, hypersensitive response (HR), activation of NADPH oxidases, ion channels and phospholipases (Suharsono et al., 2002; Torres et al., 2013). However, the phytohormonal signaling mechanisms in G-protein mediated defense responses have not yet been clearly understood. While Trusov et al. (2006) claimed that jasmonic acid (JA) was involved in heterotrimeric G-protein mediated resistance against necrotrophic pathogen in *Arabidopsis*, Trusov et al. (2009) later demonstrated that G-protein mediated resistance to necrotrophic pathogens includes mechanisms independent of salicylic acid (SA), jasmonic acid/ethylene (JA/ET), and abscisic acid (ABA) signaling. Interestingly, no demonstrable evidence is available regarding involvement of G-protein in the defense response to an obligately surviving fungal biotrophic pathogen. Moreover, there is no report on the influence of phytohormonal signaling on G-protein mediated defense response against an obligately biotrophic fungus.

Earlier we demonstrated that phytohormonal signaling can be influenced by plant associated rhizospheric microbes. We reported higher accumulation of phenolics in pea (*Pisum sativum*) leaves against infection by the obligate pathogen *Erysiphe pisi* in fluorescent *Pseudomonas* treated plants (Singh et al., 2002) and antioxidant activities against *Sclerotinia sclerotiorum* challenge in fluorescent *Pseudomonas* and *Trichoderma* treated pea (Jain et al., 2012). Under natural environmental conditions such interactions are common and therefore needs to pay attention while evaluating plant-pathogen interactions. To address the issues (i) whether G-protein signaling is involved in plant–fungal biotrophic pathogen interactions and (ii) if so, how it is influenced by common rhizosphere microbial occupants, in the present study we used pea and *E. pisi* as model system and observed the activation patterns of all three subunits of G-proteins in pea upon inoculation with the biotroph. Further, to get an idea whether soil inhabiting rhizosphere microbes influence the host G-protein activation during the interaction, we used two compatible strains one each of fluorescent *Pseudomonas* and *Trichoderma* for treating the pea seeds. Results from the present study thus believed to add a new dimension to our understanding regarding the plant–fungal biotrophic pathogen interaction and the role of heterotrimeric G-proteins in this interaction particularly in an environment partially mimicking the natural soil conditions with the use of two soil inhabiting rhizosphere microbes.

## Materials and Methods

### Experimental Setup and Conidial Germination Test

Seeds of pea (cv. AP3) were bio-primed with *Trichoderma asperellum* T42 and *Pseudomonas fluorescens* OKC according to Singh et al. (2013) individually as well as in combination. ITS region of both the microbes were sequenced and the sequences were submitted in the NCBI GenBank with the accession numbers JN128894 (*T. asperellum*) and JN128891 (*P. fluorescens*). Cell suspension of OKC was adjusted to 1.6 × 10^8^ cfu mL^-1^ whereas T42 to 2 × 10^6^ cfu mL^-1^. Primed seeds were sown in sterilized soil with vermiculite in ratio of 2:1 and ten replicates of each treatment were placed. Plants were kept under 16 h light/8 h dark in a greenhouse at 21°C. After 21 days of sowing, plants were inoculated with conidia of *E. pisi* by dusting with the help of fine brush from earlier diseased plants (Singh et al., 2002). After pathogen inoculation leaf samples were collected after 24 h for conidial germination test and for gene expression analysis by quantitative RT-PCR. Disease severity (DS) of *E. pisi* was calculated after 2 weeks of inoculation according to a 0–4 scale (Fondevilla et al., 2006), where 0 = no visible sign of disease and 4 = well developed, freely sporulating colonies. The DS was scored as the percentage of leaf coverage by the mycelium using the formula:

$$DS(\%)=\frac{Sum\,of\,ratings\,(0-4)\times 100}{Maximum\,possible\,score\,\times Total\,no.\,of\,leaves\,exa\,\min ed}$$

### Conidial and Stomata Behavior Study

Ethanol, acetic acid solution in the ratio of 3:1 was used for removal of chlorophyll from the leaves and after clearing the leaves staining was done with Coomassie blue (0.01% in methanol; Schlicht and Kombrink, 2013) for visualization of *E. pisi* conidia on leaves. After staining, leaves were kept on glass slide and observed under compound light microscope for conidial germination. Three replicates were taken from each treatment and 50 conidia were observed for germination, number of appressoria/conidia produced, number of germ tubes/conidia produced and length of the longest germ tube. Stomata behavior (50 stomata from each treatment) was also observed under a light microscope of the same leaves after 24 h of pathogen inoculation.

### Extraction and Measurement of Total Phenolics

Total phenol content (TPC) was estimated according to the Graham (1992). Briefly, fresh leaves (1 g) were separately extracted (50% aqueous methanol, thrice from each treatment), supernatant was evaporated to dryness, dissolved in distilled water (1 ml), and analyzed with a spectrophotometer (Bausch and Lomb, USA) at 700 nm by the Prussian Blue method. A standard curve was prepared from gallic acid and TPC was calculated in terms of gallic acid equivalents.

### Qualitative and Quantitative Estimation H_2_O_2_

Histochemical staining for visual analysis of H_2_O_2_ accumulation in leaves was done by 3,3-diaminobenzidine (DAB) according to Thordal-Christensen et al. (1997). H_2_O_2_ reacts with DAB to form a reddish-brown color on leaves. All leaves (treated and untreated) were incubated at room temperature in dark for 20 h with 1 mg ml^-1^ DAB solution (pH 7.5). After incubation leaves were boiled in a solution containing alcohol and lactophenol (2:1) for 5–10 min and rinsed thrice with 50% ethanol for chlorophyll removal. For quantitative analysis of H_2_O_2_ 0.1 g leaf sample from each of the treatments was crushed in an ice bath with 2.0 ml of 0.1% (w/v) of trichloroacetic acid (TCA). The crushed material was centrifuged at 12,000 × *g* for 10 min and 0.5 ml of the supernatant was taken for further process. 10 mM potassium phosphate buffer (pH 7.0) and 1 ml of 1 M potassium iodide solution was added to previous solution and incubated at room temperature for 5 min. The oxidation product formed was measured spectrophotometrically at 390 nm (Velikova, 2000). The amount of H_2_O_2_ formed was determined by correlating with the standard curve made with known concentrations of H_2_O_2_ and expressed as nmol H_2_O_2_ g^-1^ fresh weight (FW).

### Peroxidase (PO) Activity Assay

One gram of leaf sample from all treatments was homogenized in 4 ml of 0.1 M phosphate buffer (pH 7.0; 4°C) and centrifuged at 12000 × *g* at 4°C for 15 min. Supernatant was taken as enzyme source. The reaction mixture contained 2.8 ml of 0.1 M phosphate buffer (pH 7), 0.05 ml of 0.018 M Guaiacol, enzyme extract 0.1 ml, lastly 1% of H_2_O_2_ added and change in absorbance was measured immediately at 420 nm at interval of 30 s for 3 min. The enzyme activity was expressed as change in the OD (optical density) per min per gram of fresh weight. The experiment was conducted according to Bergmeyer and Bernt (1974).

### RNA Extraction and Quantitative RT-PCR Analysis

Relative expression levels of G protein subunits, *LOX1, COI1, PAL*, and *PR1* were determined by quantitative real-time PCR (Q-RT-PCR) according to Webling and Panstruga (2012). Total RNA was extracted from 200 mg leaf tissue thrice from each treatment by using RNeasy plant mini kit (Qiagen). Amount of RNA was analyzed by NanoDrop 2000 (Thermo). Approximately 3 μg total RNA was digested using RNase-free DNase I at 37°C to remove remaining genomic DNA. cDNA was prepared by following the protocol (Sambrook and Russell, 2001) with help of oligo (dT) primers and reverse transcriptase enzyme. cDNAs were used as templates for semi-quantitative and quantitative RT-PCR. Eva Green SYBR^®^ Green Supermix Kit (Bio-Rad) on the iQ5 Real-Time PCR Detection System (Bio-Rad Laboratories, Munchen, Germany) was used for qRT-PCR. We used gene-specific primers at a final concentration of 0.1 μM and transcript levels of each mRNA were determined and normalized with the level of ubiquitin. Following primers were used: Gα1 (forward: 5′-TGCCCGTGGTAATGAGCTCCA-3′, reverse: 5′-TCTCATTTCTCTGGCCGCCGA-3′), Gα2 (forward: 5′-CCGATGAGAGCAAGGACGTT-3′, reverse: 5′-GGAGTTCATTACCACGGGCA-3′), Gβ (forward: 5′-CGATGCGGACGTCAAAGAACGTC-3′, reverse: 5′-CCTTTCCGGTATGACCTTGGAGCG-3′), Gγ (forward: 5′-CGCCTGCGCCATAGACATCGT-3′, reverse: 5′-TCCAAAGTCCCCGTCGCGC-3′), *LOX1* (forward: 5′-GCTGCAGGCATGGTGGACAGA-3′, reverse: 5′-GCCCAAAGTTCACAGCTGCATGG-3′), *PR1* (forward: 5′-GCTGCCTTGTCCTTCCTCTTCC-3′, reverse: 5′-AGTGCGCTTTGTTCTTGCAGTG-3′), *COI1* (forward: 5′-AGATGTGGATGGTGTTGTTTCCCA-3′, reverse: 5′-CGGTTATCTTCTCCTCACGGTCG-3′), *PAL* (forward: 5′-ATGGTGTGAAGGTGGAGCTGTCA-3′, reverse: 5′-CGCCTTGTTTGGTTCGACGGT-3′), and Ubiquitin (forward: 5′-CCCCCAGACCAGCAAAGGTTGA-3′, reverse: 5′-TGTGTCTGAGCTCTCCACCTCCA-3′). The primers were designed by using online software Primer-3 with parameters according to Thornton and Basu (2011). qRT-PCR assays were carried out according to the modified protocol of Marone et al. (2001) in three technical replicates per sample following the conditions: denaturation at 95°C for 2 min, 40 repeats at 95°C for 20 s, 60°C for 30 s, and 72°C for 25 s. The data from different PCR runs or cDNA samples were normalized with mean CT value of the endogenous gene ubiquitin using the 2^-ΔΔCT^ method (Schmittgen and Livak, 2008). Fold accumulation of transcripts was compared by using the mean of the CT values of the three biological replicates with treatment C (control).

### HPLC Analysis of Salicylic Acid (SA) and Abscisic Acid (ABA)

HPLC (high performance liquid chromatography) was done from leaf samples collected after 24 h of pathogen inoculation. The samples were prepared according to method of Basha et al. (2006). 1 g of sample was homogenized in 10 ml of ethanol water (4:1) and centrifuged at 13000 rpm for 15 min at room temperature. Greenish supernatant was taken and the green pigments were removed by adding small amount of charcoal step by step. After 3 h, clear transparent solution was filtered through Whatman No. 1 filter paper and collected in glass tube, the process is repeated two times. The supernatant was evaporated and dried samples were resuspended in HPLC grade methanol for HPLC analysis. Shimadzu LC-10A (Japan) was used that was equipped with dual pump LC-10A binary system, UV detector SPD-10A, Phenomenex (Torrance, USA) C18 column (RP-Hydro, 4 μm, 250 mm × 4.6 mm). Shimadzu Class VP series software was used to integrate the data. Separation of SA was achieved with acetonitrile/water (1:1 v/v) containing 1% acetic acid in a linear gradient program (Singh et al., 2009). The solvent flow rate was 1.0 ml min^-1^. Results (μg/g) fresh weight FW were obtained by comparing the peak areas of the samples with standards. For ABA, solvent system and running conditions were set according to Poschenrieder et al. (1989).

### Statistical Analysis

Statistical analysis was done by using SPSS version 16. Experiments were repeated two times using a completely randomized design. The data are expressed as the mean of three independent replications ± standard deviation. The treatment mean values were compared by Duncan’s multiple range test at *P* ≤ 0.05 significance level.

## Results

### Transcript Accumulation of Subunits of Pea G-protein Pre-treated with OKC and T42 and Challenged with *Erysiphe pisi*

**FIGURE 1:**
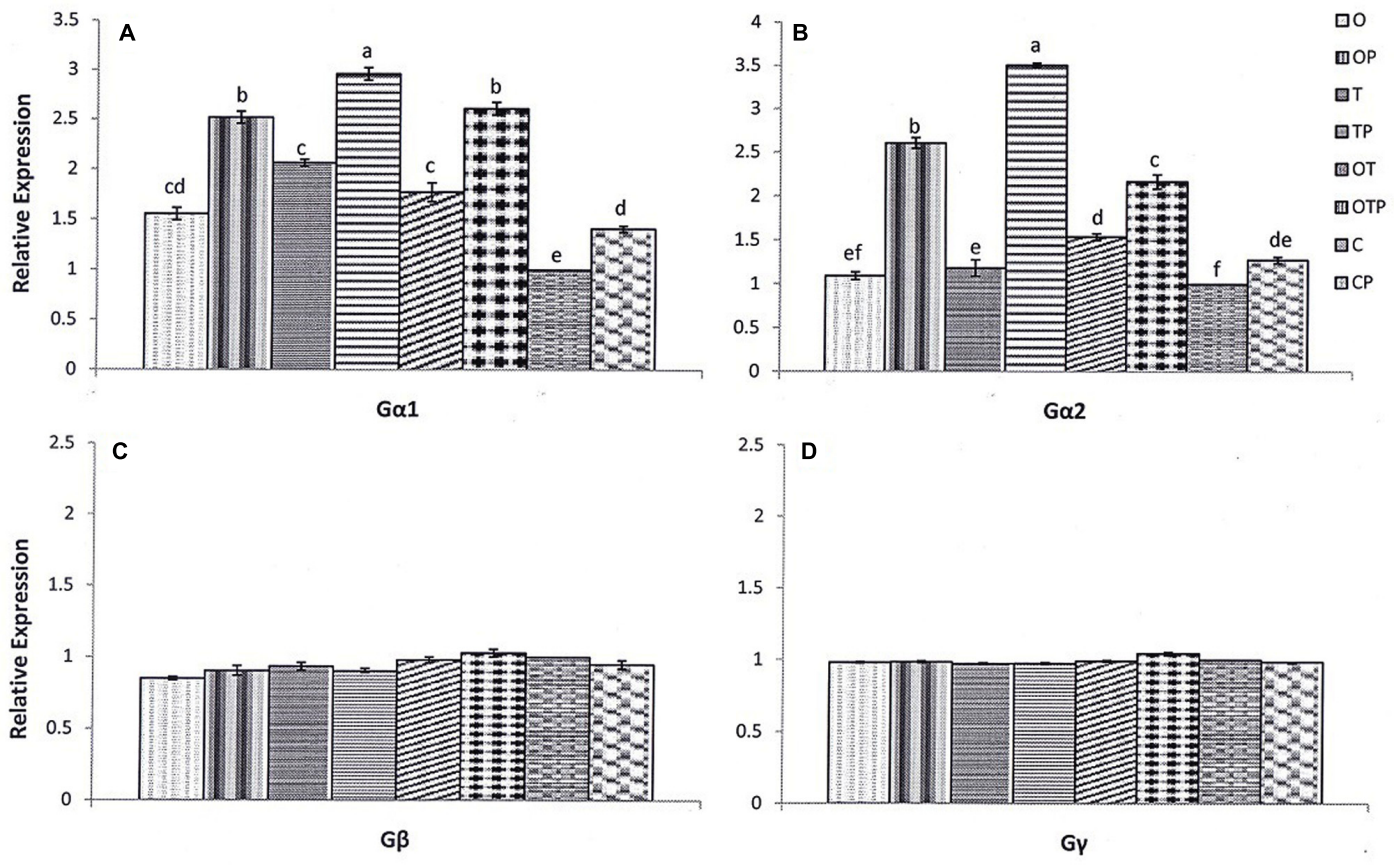
**Relative transcript accumulation pattern of pea heterotrimeric G-protein subunits [Gα1 **(A)** and 2 **(B)**, Gβ **(C)** and Gγ **(D)**] as analyzed through quantitative real time-PCR in different treatments after 24 h of pathogen (*Erysiphe pisi*) challenge: O- *Pseudomonas fluorescens* OKC; OP- OKC treated and pathogen challenged; T*- Trichoderma asperellum* T42; TP- T42 treated and pathogen challenged; OT- combination of OKC and T42; OTP- OKC and T42 treated and pathogen challenged; P- Pathogen challenged and C- OKC and T42 non-treated and pathogen unchallenged control.** The qRT-PCR data were normalized with mean CT value of the endogenous gene ubiquitin and fold accumulation of transcripts was compared by using the mean of the CT values of the three biological replicates with treatment C (control). Error bars represent SD (Standard Deviation) from means of three measurements. Different superscript letters indicate data significantly different from the other treatments (*P* ≤ 0.05; Duncan’s multiple range test).

Differential responses of the heterotrimeric G-protein subunits are reported against a variety of pathogens including necrotrophic fungi, vascular pathogens, oomycete, and bacterial pathogens (Trusov et al., 2010). In the present study we observed responses of all three subunits of the pea heterotrimeric G-protein pre-treated with *P. fluorescens* OKC and *T. asperellum* T42 followed by challenge with the biotrophic pathogen *E. pisi.* Quantitative real time PCR analyses proved that it was mostly transcripts of Gα-subunits (Gα1 and 2) of the G-protein accumulated after the biotroph challenge particularly in treatment T42 (**Figures 1A,B**). The results authenticate a positive role of the microbes in stimulating Gα (1, 2) transcript accumulation. The Gα1 transcript accumulation after the pathogen challenge was approximately threefold more over unchallenged control in the T42 treatment followed by nearly 2.8 folds more in the co-inoculated treatment and 2.5 folds in treatment OKC. The transcript level of Gα1 was lowest in the unchallenged control compared to the pathogen challenged plants. A similar trend was observed in Gα2 transcript accumulation where its transcript accumulation was nearly 3.5 folds in T42, 2.5 folds in OKC and 2.2 folds in the co-inoculated treatment. Between the two introduced microbes *T. asperellum* T42 performed better compared to *P. fluorescens* OKC. In contrast, the transcript levels of the Gβ and Gγ-subunits were either basal or lower in most of the treatments compared to control plants (**Figures 1C,D**). However, weak expressions of Gγ were observed in the pathogen challenged plants pre-treated either with OKC or co-inoculated with OKC and T42.

### JA-Mediated Defense Responses in Pea Pre-treated with OKC and T42 followed by *E. pisi* Challenge

**FIGURE 2:**
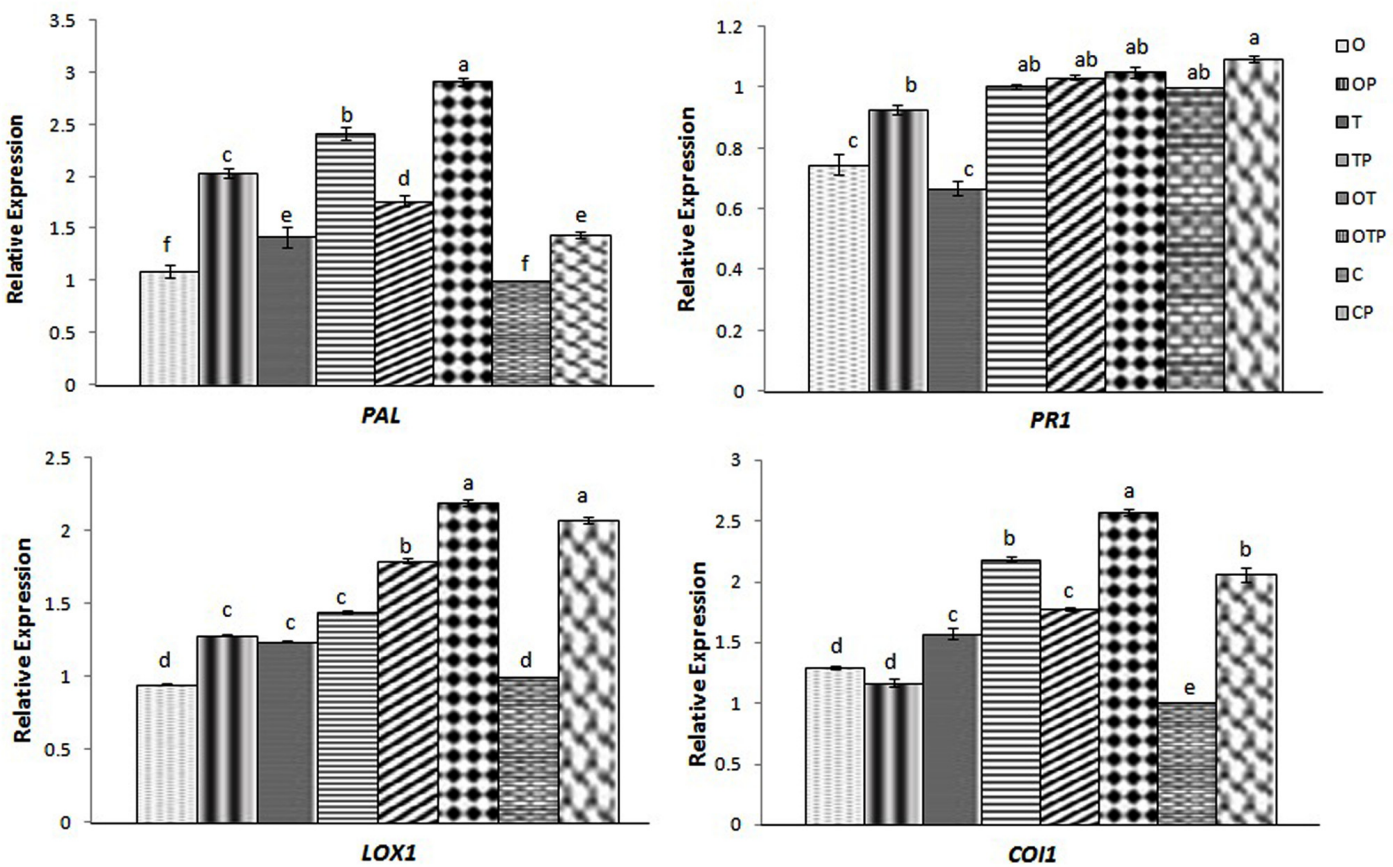
**Relative transcript accumulation patterns of two key genes representing JA (*LOX1* and *COI1*) and SA (*PAL* and *PR1*) -mediated defense responses in pea leaves during challenge by the biotroph fungal pathogen *Erysiphe* as analyzed through quantitative real time-PCR in different treatments after 24 h of pathogen (*E. pisi*) challenge: O- *P. fluorescens* OKC; OP- OKC treated and pathogen challenged; T*- T. asperellum* T42; TP- T42 treated and pathogen challenged; OT- combination of OKC and T42; OTP- OKC and T42 treated and pathogen challenged; P- Pathogen challenged and C- OKC and T42 non-treated and pathogen unchallenged control.** The qRT-PCR data were normalized with mean CT value of the endogenous gene ubiquitin and fold accumulation of transcripts was compared by using the mean of the CT values of the three biological replicates with treatment C (control). Error bars represent SD from means of three measurements. Different superscript letters indicate data significantly different from the other treatments (*P* ≤ 0.05; Duncan’s multiple range test).

**FIGURE 3:**
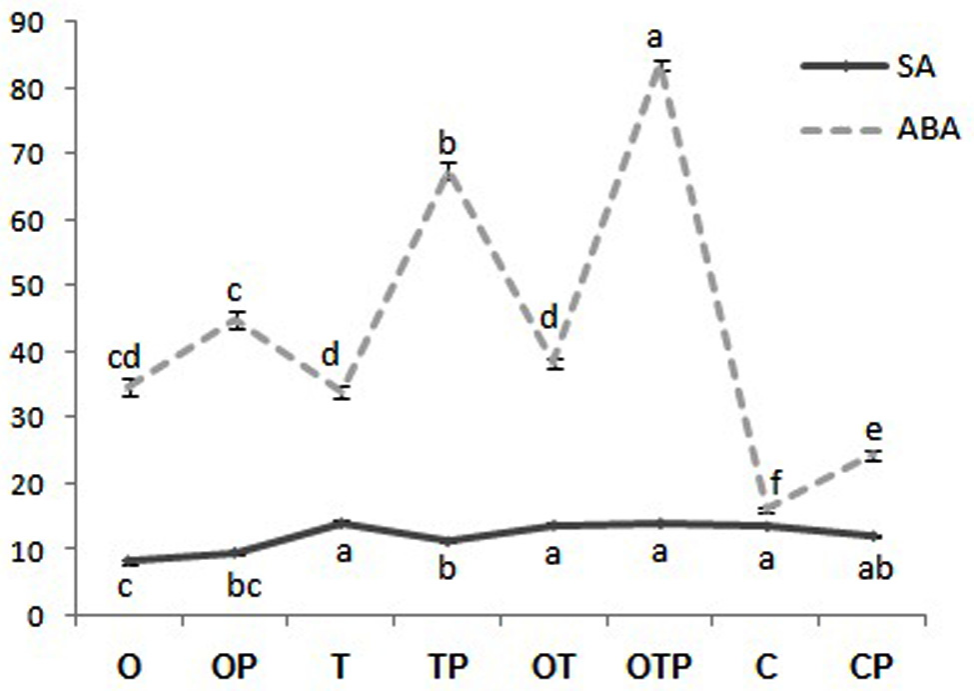
**Relative abundance of salicylic acid (SA) and abscisic acid (ABA) in pea leaves after 24 h of pathogen (*Erysiphe pisi*) challenge in different treatments: P- Pathogen *E. pisi*, O- *P. fluorescens* OKC, T- *T. asperellum* T42, OT- combination of OKC and T42.** Error bars represent SDs from means of three measurements. Different superscript letters indicate data significantly different from the other treatments (*P* ≤ 0.05; Duncan’s multiple range test).

Jasmonic acid-mediated signal transduction has traditionally been linked to infection by necrotrophic pathogens (Thomma et al., 1998; van Wees et al., 2003; Glazebrook, 2005; Kachroo and Kachroo, 2009) whereas SA to biotrophic pathogens (Spoel et al., 2007). In contrast, non-activation of the SA pathway is also reported to infection by some biotrophic pathogens (Murray et al., 2007; Fabro et al., 2008) and instead there are reports where regulation of the JA pathway was demonstrated upon infection by biotrophic pathogens (Ellis et al., 2002). In the present study we also found that combined application of OKC and T42 increased the transcript level of one of the JA-biosynthetic pathways genes *LOX1* in pea during infection by the biotrophic pathogen *E. pisi* (**Figure 2**). Interestingly its accumulation was not significantly high compared to control when OKC and T42 applied individually. Rather the co-inoculation effect was nearly at par with the only pathogen challenged plants without any microbial inoculations. A similar trend was also observed with the transcript accumulation pattern of the JA receptor *COI1* (Yan et al., 2009). Its accumulation was also high in combined application of OKC and T42 in presence of the pathogen as well as in only pathogen challenged plants (**Figure 2**). Therefore, relatively higher activations of *LOX1* and *COI1* in *E. pisi* challenged pea plants co-inoculated with OKC and T42 and only *E. pisi* challenge may mainly be due to the pathogen activities. However, the role of T42 in strengthening transcription of the two genes also cannot be ignored and between the two microbes certainly the role of T42 was more compared to OKC. Further, basal level activation of the *PR1* (**Figure 2**), a marker gene in the SA-mediated signaling pathway, as well as basal level or lower accumulation of SA (**Figure 3**) in all treatments confirms non-regulation of the SA-signal transduction pathway in any of the treatments (**Figure 2**). Although, transcripts of *PAL* were higher in co-inoculation of OKC and T42, its accumulation may be attributed to activation of the phenylpropanoid pathway (Singh et al., 2013) leading to biosynthesis of phenolics (**Figure 6** rather than SA, **Figure 3**).

### H_2_O_2_ Generation and Peroxidase Activities in Pea Pre-treated with OKC and T42 followed by *E. pisi* Challenge

**FIGURE 4:**
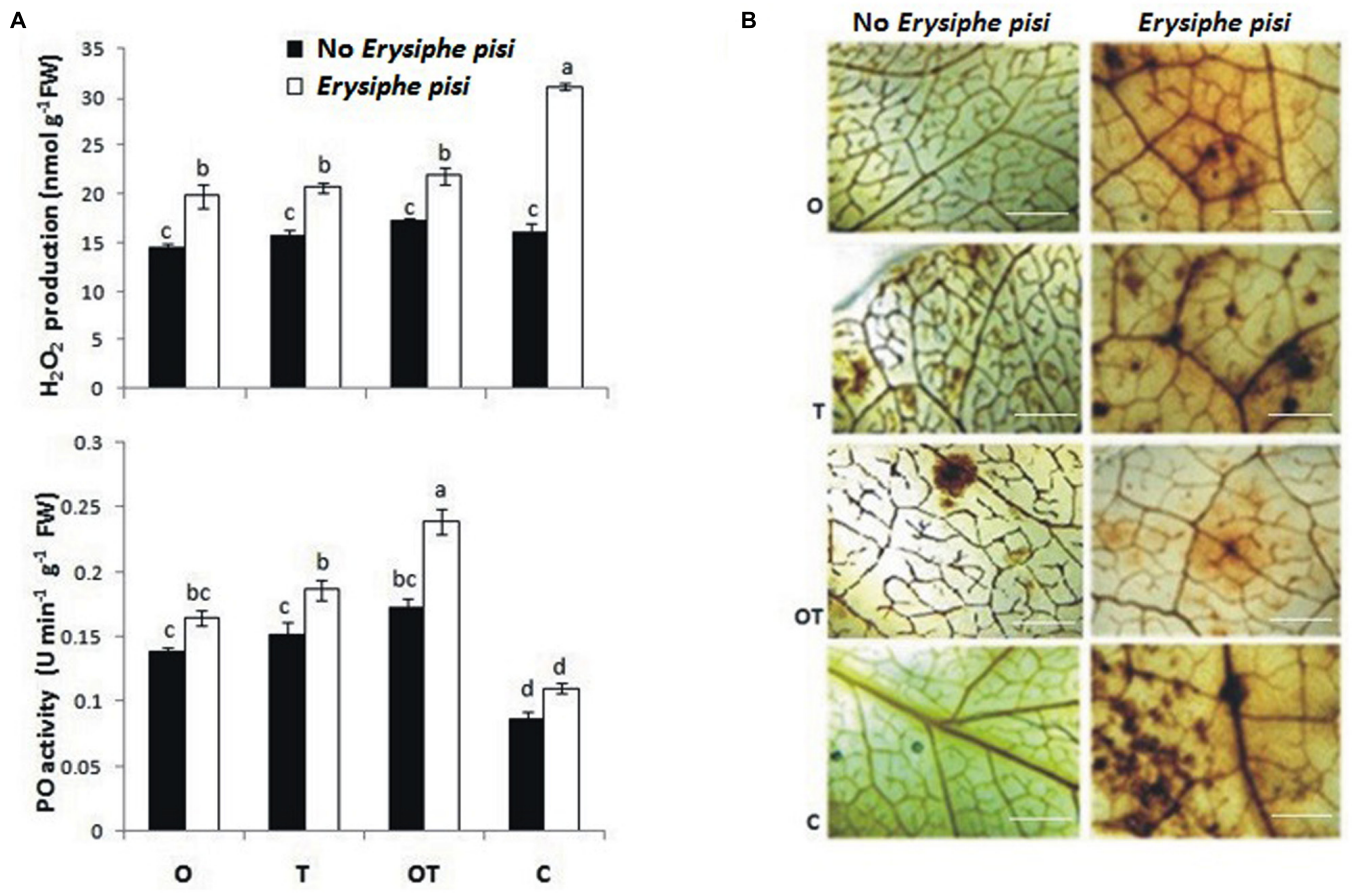
**Reactive oxygen species [hydrogen peroxide (H_2_O_2_)] generation and antioxidant [peroxidase (PO)] activities in pea leaves modulated by *P. fluorescens* OKC and *T. asperellum* T42 during challenge by the biotroph fungal pathogen *E. pisi* as analyzed through spectrophotometrically and histochemical staining.** **(A)** H_2_O_2_ accumulation and PO activities in pea leaves raised from different treatments: O- *P. fluorescens* OKC, T*- T. asperellum* T42, OT- combination of OKC and T42, C- Control after 24 h of *E. pisi* challenge. Error bars represent SD from means of three measurements. Different superscript letters indicate data significantly different from the other treatments (*P* ≤ 0.05; Duncan’s multiple range test). **(B)** Histochemical staining confirms biochemical estimation of H_2_O_2_ in leaves raised from same treatments as in **(A)**. Brown spots represent accumulation of H_2_O_2_. The bars are equivalent to 100 μm.

In plant cells the heterotrimeric G-proteins also regulate ROS metabolism (Zhao et al., 2010). Accumulation of H_2_O_2_ takes place due to mediation by G-proteins during stomata movement and the phytohormone ABA is involved in the process (Zhang et al., 2011). Among the three subunits of G-proteins the role of Gα-subunit had been established in mediating the process of ROS accumulation (He et al., 2013). We found that H_2_O_2_ production was significantly high in *E. pisi* challenged leaves but low in microbial treatments even after pathogen challenge (**Figure 4A**). DAB staining also confirmed the same observation (**Figure 4B**). Interestingly, the pattern of H_2_O_2_ production also coincides with ABA accumulation in the same treatments (**Figure 3**). Production of H_2_O_2_ targeting the fungal hypha of *Colletotricum graminicola* in maize during the early biotrophic growth of the pathogen was demonstrated (Vargas et al., 2012) and the phenomenon is confirmed in the present study. Results of the present study clearly establish an interrelationship between activation of Gα (1, 2)-subunits of the G-protein with H_2_O_2_ and ABA accumulation in pea pre-treated with OKC and T42 and under the challenge of *E. pisi*. Lower manifestations of H_2_O_2_ levels in microbial treatments can be explained through the antioxidant mechanism of the host which was highly active in those treatments. PO activities in the microbial treatments were very high particularly in the co-inoculated treatment. The trends of H_2_O_2_ accumulation and PO activities were the same and correlated with each other. The observation indicates a positive correlation between Gα (1,2)-mediated signal transduction, ROS generation and PO activities in pea particularly under the influences of OKC and T42 after challenge with *E. pisi*.

### Stomata Behavior in Pea Leaves Pre-treated with OKC and T42 followed by *E. pisi* Challenge

**FIGURE 5:**
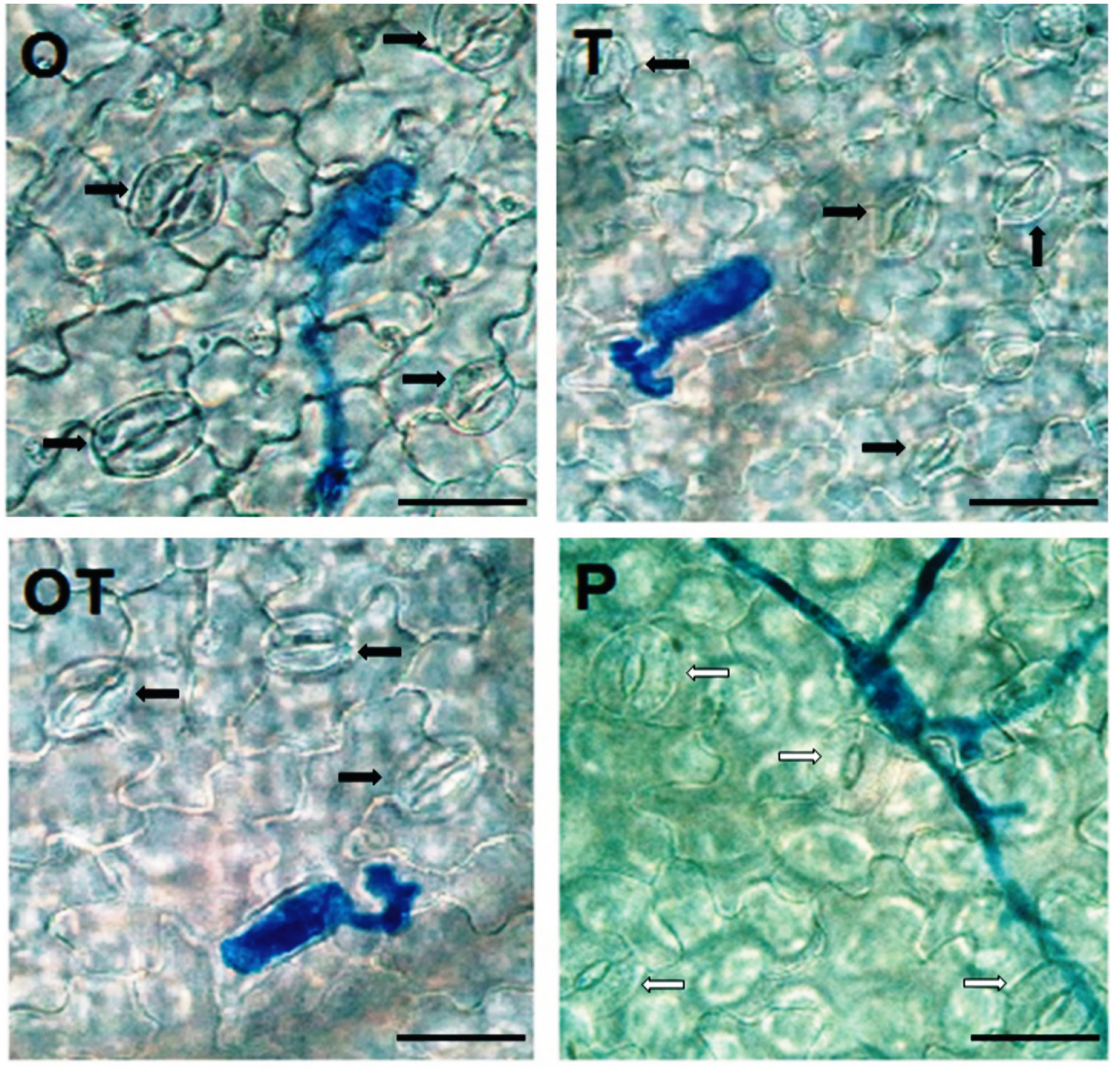
**Stomata behavior in pea leaves after 24 h of pathogen (*E. pisi*) challenge in different treatments: P- Pathogen *E. pisi*, O- *P. fluorescens* OKC, T*- T. asperellum* T42, OT- combination of OKC and T42.** Stomata of pea leaves were open in treatments P and O, nearly closed in T and closed in OT. White arrows indicate open stomata and black arrows indicate closed stomata. The bars are equivalent to 100 μm.

**FIGURE 6:**
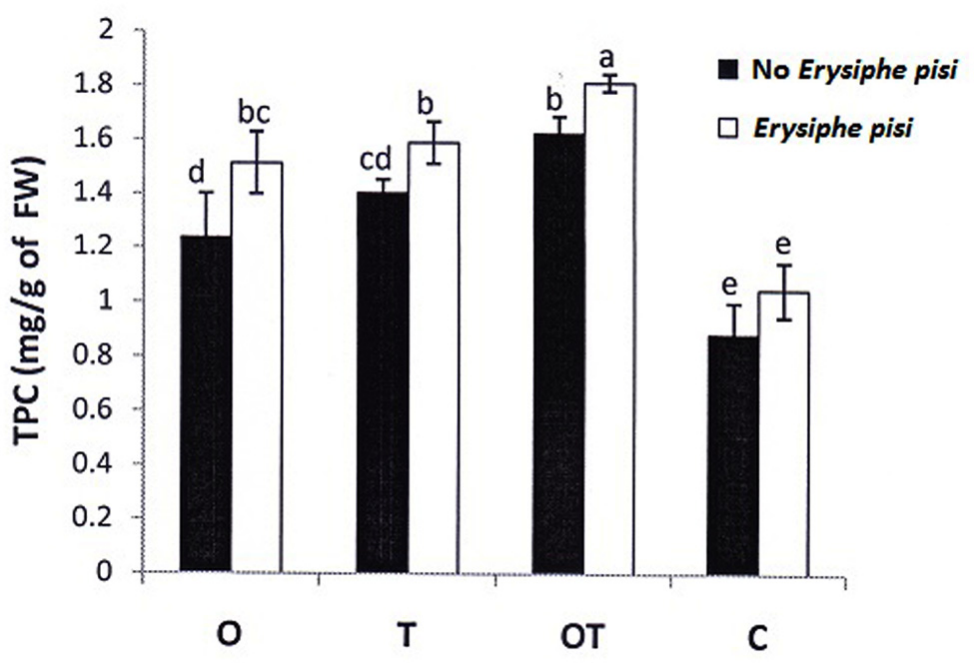
**Total phenol content in pea leaves following challenge by *E. pisi* in different treatments: O- *P. fluorescens* OKC, T*- T. asperellum* T42, OT- combination of OKC and T42, C- Control.** Error bars represent SD from means of three measurements. Different superscript letters indicate data significantly different from the other treatments (*P* ≤ 0.05; Duncan’s multiple range test).

Stomata behavior in hosts infected by biotrophic pathogens had been recorded in several instances. Stomata opened more widely in barley and pea leaves infected by the biotroph *E. graminis* f.sp. *hordei* and *E. pisi*, respectively in the early stages of infection (Graf-Marin, 1934; Ayres, 1976). In the present study also we found that stomata remained open in the early period of infection, i.e., after 24 h of inoculation with the biotroph conidia (**Figure 5**). However, the stomata remained nearly closed in the microbe treated plants after 24 h. The nearly closed stomata in microbes treated pea leaves opposed to open stomata in only pathogen challenged pea closely reflect the Gα (1 and 2) subunits - activation pattern in the same treatments (**Figures 1** and **5**). External stimuli induced and Gα-mediated stomata closure was reported earlier by Chen et al. (2004) and recently by He et al. (2013). Interestingly in both the cases Gα-regulated stomata closure was mediated by H_2_O_2_. A similar relationship of H_2_O_2_ generation and stomata behavior was also observed in the present investigation. PO activities in the microbial treatments must also be considered while considering H_2_O_2_ generation in the same treatments. Partial or complete closure of stomata in the present investigation after inoculation with the biotrophic pathogen thus can be clearly correlated with H_2_O_2_ generation and Gα (1, 2) transcript accumulation. Moreover, stomata closure was also linked to phenolics accumulation (Plumbe and Willmer, 1986). In the present study we also observed that a positive correlation exists between phenolics accumulation and stomata closure. We observed that in leaves where phenolics accumulation was higher stomata closure was also high (**Figure 6**). The activation pattern of Gα subunits, accumulation of H_2_O_2_ and phenolics and stomata behavior positively correlates with each other in the present study and all three activities was enhanced in plants pre-treated with the soil inhabiting rhizosphere microbial strains. The contrasting behavior of stomata after pathogen inoculation in the microbe co-inoculated plants may thus be postulated through Gα-regulated responses.

### Development of *E. pisi* on Pea Pre-treated with OKC and T42

**FIGURE 7:**
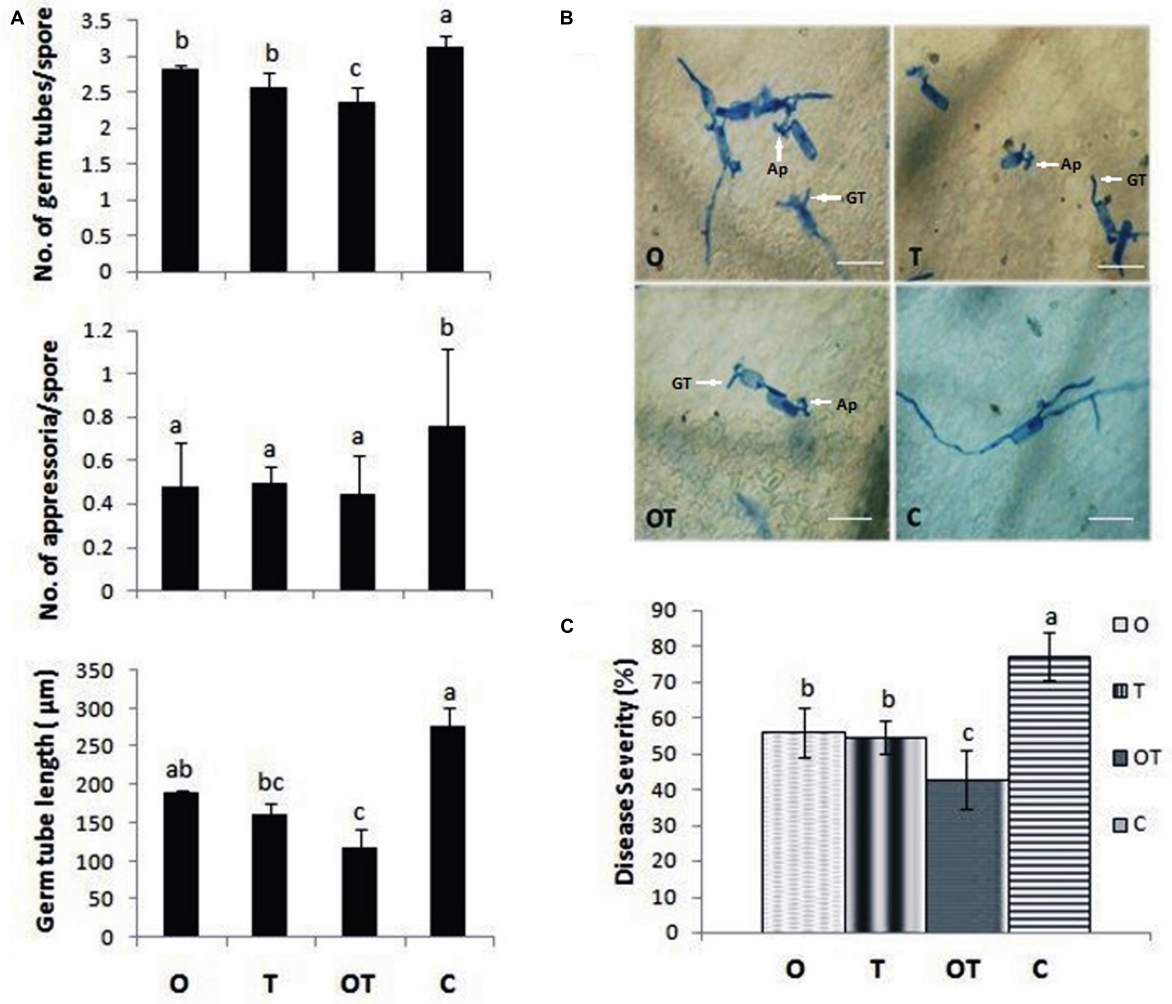
***Erysiphe pisi* conidia growth and disease development on pea leaves.** **(A)** Inhibition of spore germination in terms of number of germ tubes/spore, number of appressoria/spore and longest germ tube length on plant leaves raised from different treatments: O- *P. fluorescens* OKC, T*- T. asperellum* T42, OT- combination of OKC and T42, C- Control after 24 h of *E. pisi* challenge. **(B)** Confirmation of **(A)** on plant leaves by histochemical staining in same treatments. GT represents germ tube and Ap represents appressorium. **(C)** Disease severity of *E. pisi* on pea plants 2 weeks after inoculation. Error bars represent SD from means of three measurements. Different superscript letters indicate data significantly different from the other treatments (*P* ≤ 0.05; Duncan’s multiple range test). The bars are equivalent to 100 μm.

Pea plants pre-treated with the soil inhabiting rhizosphere microbes and challenged with *E. pisi* demonstrated genetic and physiological similarities with an enhanced degree of the Gα-regulated responses. Coinciding with the genetic and physiological outcome, development of *E. pisi* on pea leaves pre-treated with the microbes also showed similar responses. Conidial germination, appressoria formation, and germ tube development were reduced significantly in leaves of pea co-inoculated with microbial strains OKC and T42 compared to their single applications and only pathogen challenged plants without pre-treatment with either OKC or T42 (**Figures 7A,B**). Further, disease severity was also reduced significantly in OKC and T42 pre-treated plants (**Figure 7C**). The reduced conidial development and disease severity of *E. pisi* in pea plants pre-treated with OKC and T42 may be attributed to the stimulus generated by the microbes. Further, the pathogen suppressive effect was possibly achieved through enhanced activities of Gα (1 and 2), H_2_O_2_ and accumulation of antimicrobial phenolics.

## Discussion

### Transcript Accumulations of G-protein Subunits

Heterotrimeric G-proteins are widely conserved in plants (Assmann, 2002). They have been implicated to be involved in diverse signaling processes related to growth, cell proliferation, defense, stomatal movements, channel regulation, sugar sensing, and responses to phytohormones (Urano et al., 2013). There is some evidence regarding involvement of plant G-proteins in stimulation of host defense mechanisms against necrotrophic and vascular pathogens. Recently, Lorek et al. (2013) demonstrated that genetic alterations of the mildew resistance locus O (MLO) or Gβ-subunit of the heterotrimeric G proteins in *Arabidopsis thaliana* increased susceptibility to the obligate bacterial pathogen *P. syringae.* However, there is lack of information regarding activation pattern of plant G-proteins when challenged with an obligately surviving fungal biotrophic pathogen and how the rhizosphere microbes influence the process. Thus, in the present study we have monitored activation patterns of all subunits [α(1,2), β, and γ] of the pea G-protein complex under the challenge of a biotrophic pathogen *E. pisi* as well as influence of two soil inhabiting rhizosphere microbes *P. fluorescens* OKC and *T. asperellum* T42 mimicking the environmental conditions partially. We found that G-protein mediated signaling was active during pea-*E. pisi* interactions and a number of evidences suggested the same. To our surprise, among the three subunits of G-proteins we found that mostly the two subunits of Gα (1 and 2) participated in the regulation of pea-*E. pisi* interaction and their activations were significantly influenced by the two soil microbes under the pathogen challenged condition. Between the two microbial strains *T. asperellum* T42 had the highest influence on accumulation of Gα (1 and 2) transcripts. The observation clearly demonstrated that pea was benefited by association of the two compatible microbial strains (OKC and T42). Earlier studies have demonstrated that plants derive highest benefit from compatible rhizosphere microbes (Barea et al., 2005; Jain et al., 2012; Singh et al., 2014). Moreover, basal or reduced activation of Gβ and weak expression of Gγ in few treatments clearly does not indicate any positive role of the Gβγ complex whereas strong expression of Gα (1 and 2) indicate a positive role of Gα (1 and 2) possibly leading to induction of defense responses during *E. pisi* challenge particularly in presence of the microbial strains OKC and T42. The results are in contrast to the results with the necrotrophs in *Arabidopsis* where Gα was reported to be a negative regulator in the G-protein mediated signaling (Trusov et al., 2006) and Gβ is a positive regulator (Llorente et al., 2005; Trusov et al., 2006). However, involvement of Gα subunit in defense signaling was demonstrated against the rice blast pathogen *M*. *grisea* in rice (Suharsono et al., 2002) but the fact of the matter is that *M. grisea* is not an obligate biotroph and therefore, host responses toward it may not be considered to be universal as each host-pathogen pair may generate different signals. Further, defense responses in monocots may alter from the dicot plants as evident from some earlier studies (Tamaoki et al., 2013). Hence, the results obtained from the present study have a clear significance in relation to understanding the plant G-protein response to an obligate fungal pathogen.

### H_2_O_2_ Generation, Phenolics Accumulation, and Stomata Behavior

The Gα (1 and 2) mediated pea defense responses was also partially manifested by the reduced development of germinated *E. pisi* conidia, lowered disease severity and closure of stomata in pathogen challenged pea leaves under influence of the rhizosphere microbes. Foliar pathogens including biotrophs like *E. pisi* can reduce water use efficiency by host plants leading to closure of stomata (Ayres, 1976; Grimmer et al., 2012). However, stomatal closure can also be contributed by accumulation of phenolic compounds (Plumbe and Willmer, 1986) and ABA (Whenham and Fraser, 1981). In the present study also total phenolics and ABA contents increased in pea when challenged with the biotroph and the levels were even higher when treated with the soil inhabiting rhizosphere microbes. Phenolics accumulation in the combined microbial treatments was supported by high transcript accumulation of the first gene of the phenylpropanoid pathway *PAL.* The partially closed stomata in microbes treated pea leaves after challenge with the biotroph compared to non-closure of stomata in only biotroph challenged pea leaves without microbial treatment point out a correlation between the role of phenolics and ABA accumulation with stomata behavior. Similarly, it was reported that Gα transmit various signals leading to stomata closure through stimulation of H_2_O_2_ production (Chen et al., 2004; He et al., 2013). We also recorded a similar observation and found that H_2_O_2_ accumulation was more in pathogen challenged leaves. However, the manifested levels of H_2_O_2_ in the results are attributed to high PO activities. The observation further strengthened the possibility of linkages between Gα (1 and 2) and H_2_O_2_ in regulating stomatal closure which was more prominent in OKC and T42 treatments.

### Phytohormonal Signaling and *E. pisi* Development

The role of phytohormones such as SA and JA/ET, normally associated with host defense responses, is not clearly understood in case of G-protein mediated signal transduction. Moreover, the phytohormonal signaling in monocots and dicots are not similar (Tamaoki et al., 2013). G-protein mediated defense responses in *Arabidopsis* when challenged with the necrotrophic pathogen *A. brassisicola* and vascular pathogen *F. oxysporum* was found to be independent of either SA or JA/ET mediation (Trusov et al., 2009). However, Okamoto et al. (2009) demonstrated that a set of JA regulated genes was also regulated by Gα subunit in *Arabidopsis*. Further, it was shown that certain biotrophic fungal species also triggers activation of JA-mediated responses which was earlier linked to suppression of JA-mediated responses (Antico et al., 2012). In the present study, relatively higher transcript accumulations of *LOX1* and *COI1* in pathogen challenged pea leaves reflect activation pattern of the Gα-subunits in the same treatments involving the rhizosphere microbes and thus a positive correlation could be established. Further, non-accumulation of SA and simply basal expression of *PR1*, a marker of SA signaling pathway, in the same treatments also confirmed non-involvement of the SA. It is believed that JA and SA signal transduction pathways are mutually antagonistic in dicotyledonous plants in most instances (Niki et al., 1998; Koornneef and Pieterse, 2008). However, this observation needs further confirmation due to existence of conflicting evidences (Thaler et al., 2012).

**FIGURE 8:**
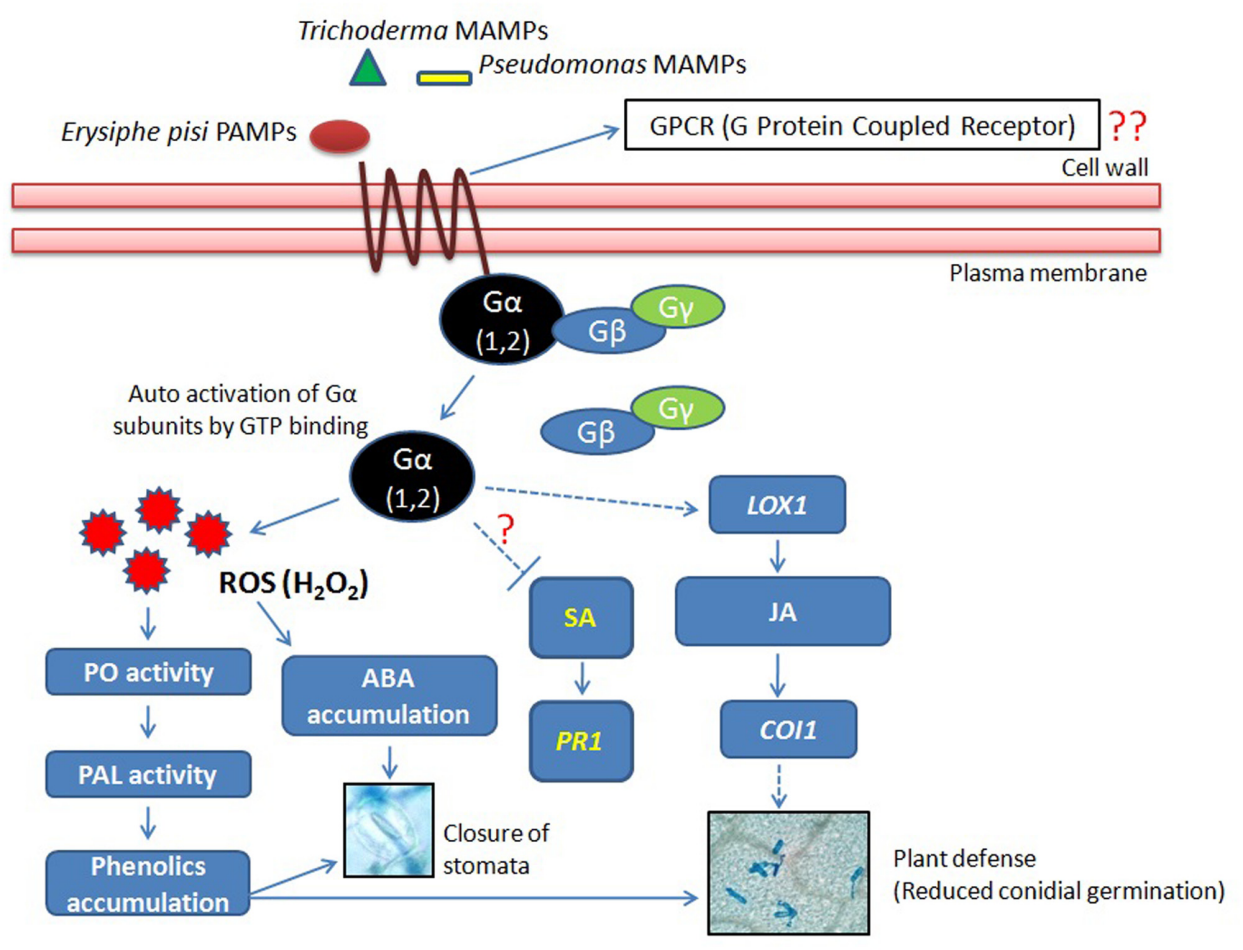
**Co-inoculation of *P. fluorescens* OKC and *T. asperellum* T42 as pretreatment stimulate transcript accumulations of Gα subunits 1 and 2 of the heterotrimeric G protein during *E. pisi* challenge.** The co-inoculated microbes also generated reactive oxygen species (ROS) H_2_O_2_ but its concentration was regulated by activities of peroxidases. ROS generation also stimulated activities of the first gene of the phenyl propanoid pathway *PAL* that perhaps led to phenol accumulation in pea during infection by *E. pisi.* The signal transduction due to activation of Gα subunits was possibly mediated through JA in pea under stimulus of the microbes and the cumulative effect of co-inoculated microbes had a suppressive effect on *E. pisi* conidial development on pea leaves.

From the results of present study, it can be concluded that the biotroph *E*. *pisi* elicited activation of the Gα (1 and 2)-subunits in pea that were further influenced by the rhizosphere microbes particularly by the *Trichoderma* strain T42 (**Figure 8**). The microbes (OKC and T42) when co-inoculated also stimulated the JA-mediated signal transduction pathway relatively more compared to only *E. pisi* challenged plants that probably led to enhanced accumulation of phenolic compounds (Gadzovska et al., 2007) and stomata closure. In summary, significant suppressive effect of the co-inoculated microbes (OKC and T42) was observed on development of *E. pisi* in pea and there was a positive correlation between transcript accumulation patterns of Gα subunits 1 and 2 with reduced *E. pisi* conidial development.

## Author Contributions

BS conceived the study, JP and BS conducted the study, BS, HS, RU, RK, and MA supervised the project, BS and HS wrote the main paper, and JP conducted the statistical analyses.

## Conflict of Interest Statement

The authors declare that the research was conducted in the absence of any commercial or financial relationships that could be construed as a potential conflict of interest.
